# Semi-Supervised Burn Depth Segmentation Network with Contrast Learning and Uncertainty Correction

**DOI:** 10.3390/s25041059

**Published:** 2025-02-10

**Authors:** Dongxue Zhang, Jingmeng Xie

**Affiliations:** 1 Changchun Institute of Optics, Fine Mechanics and Physics, Chinese Academy of Sciences, Changchun 130033, China; zhangdongxue@ciomp.ac.cn; 2 College of Electronic Information, Xi’an Jiaotong University, Xi’an 710049, China

**Keywords:** semi-supervised, burn depth segmentation, contrastive learning, uncertainty correction

## Abstract

Burn injuries are a common traumatic condition, and the early diagnosis of burn depth is crucial for reducing treatment costs and improving survival rates. In recent years, image-based deep learning techniques have been utilized to realize the automation and standardization of burn depth segmentation. However, the scarcity and difficulty in labeling burn data limit the performance of traditional deep learning-based segmentation methods. Mainstream semi-supervised methods face challenges in burn depth segmentation due to single-level perturbations, lack of explicit edge modeling, and ineffective handling of inaccurate predictions in unlabeled data. To address these issues, we propose SBCU-Net, a semi-supervised burn depth segmentation network with contrastive learning and uncertainty correction. Building on the LTB-Net from our previous work, SBCU-Net introduces two additional decoder branches to enhance the consistency between the probability map and soft pseudo-labels under multi-level perturbations. To improve segmentation in complex regions like burn edges, contrastive learning refines the outputs of the three-branch decoder, enabling more discriminative feature representation learning. In addition, an uncertainty correction mechanism weights the consistency loss based on prediction uncertainty, reducing the impact of inaccurate pseudo-labels. Extensive experiments on burn datasets demonstrate that SBCU-Net effectively leverages unlabeled data and achieves superior performance compared to state-of-the-art semi-supervised methods.

## 1. Introduction

Burns are a common type of traumatic injury caused by factors such as heat, electricity, and chemicals. According to the World Health Organization, approximately 11 million burn incidents occur globally each year, with around 180,000 resulting in fatalities [1]. One of the most critical threats to burn patients is infection, particularly in cases of deep burns where infections can lead to severe complications and even pose life-threatening risks [2,3,4]. The timely and accurate diagnosis of burn depth is therefore essential for preventing infections and saving patient lives [5,6].

In clinical practice, burn depth is commonly categorized into four levels based on the three-degree, four-category classification system: superficial (first degree), superficial partial thickness (second degree), deep partial thickness (second degree), and full thickness (third degree) [7]. Current methods for diagnosing burn depth primarily rely on a physician’s visual assessment of factors such as wound appearance, moisture level, and sensitivity to external stimuli. However, this approach heavily depends on the physician’s personal experience, introducing significant subjectivity and limiting the method’s accuracy to 60–70% [8]. Instrument-based diagnostic methods, including laser Doppler imaging [9], infrared imaging [10], and laser speckle imaging [11], provide higher accuracy. However, these methods require expensive and complex equipment, limiting their accessibility and widespread clinical application.

With advancements in computer vision applied to natural images, an increasing number of researchers are exploring its applications in burn diagnosis to assist clinicians with early burn depth assessment. For example, Chang et al. [12] conducted experiments with models such as U-Net [13], PSPNet [14], DeepLabV3+ [15], and Mask R-CNN [16], demonstrating promising performance in distinguishing between burn and non-burn areas. Liu et al. [17] further integrated HRNet [18] to propose an end-to-end framework named HRNetV2-C1 for burn region segmentation and burn depth diagnosis. This model not only segmented burn and non-burn areas, but it also attempted to differentiate between varying burn depths. However, segmentation algorithms based on supervised learning require a large volume of high-quality, pixel-level labeled data for training. Due to the diverse morphology and varying sizes of burns, there is a strong demand for comprehensive and diverse datasets. Moreover, precisely annotating burn images, which is typically performed by experienced burn specialists, requires significant expertise and extensive clinical experience. This annotation process is not only time consuming, but also costly. The complexity of annotation is further exacerbated in images with indistinct burn edges or varying burn depths.

In the field of medical image segmentation, researchers are increasingly turning to semi-supervised learning methods to address data scarcity and the difficulty of data annotation. Current semi-supervised approaches primarily focus on consistency regularization techniques. For instance, Mean Teacher [19] and MC-Net [20] utilize image perturbation and network perturbation, respectively, to minimize inconsistencies in predictions for unlabeled data. Similarly, UA-MT [21] estimates uncertainty through Monte Carlo dropout in the Mean Teacher framework, and it filters unreliable predictions using predefined thresholds. Despite their advancements, these methods face several limitations, making their direct application to burn diagnosis challenging. First, existing consistency regularization approaches often rely on single-level perturbations, either at the image or network level, which limits their ability to capture feature-level information and reduces robustness. Second, burn image edge regions are particularly challenging to segment accurately, and current methods do not explicitly model these distinctive features, resulting in imprecise edge predictions. Furthermore, the lack of labeled data makes predictions on unlabeled data prone to error. While UA-MT attempts to filter unreliable predictions via multiple forward passes, this process increases training redundancy and relies on empirically determined thresholds, which are difficult to adapt to the burn domain.

To tackle these challenges, we introduce SBCU-Net, a semi-supervised burn depth segmentation network with contrastive learning and uncertainty correction. This network leverages unlabeled data and builds upon our previous work, LTB-Net [22]. SBCU-Net introduces two additional decoders to generate multiple perturbations through feature direct concatenation and edge enhancement methods, employing consistency regularization to enforce prediction consistency. To enhance segmentation performance in edge regions, we integrate a contrastive learning strategy to align and strengthen predictions from each branch decoder. Furthermore, an uncertainty correction mechanism is designed to reduce the effect of unreliable predictions, thereby refining the training process and improving the effectiveness of the semi-supervised model. Our framework outperforms current semi-supervised methods, enhancing its application to burn depth segmentation. The primary contributions of this study are as follows:

(1) We introduce two additional decoders based on our previous work LTB-Net [22] to form a three-branch decoder structure. One decoder concatenates features directly to introduce network perturbations, while the other employs feature edge enhancement to introduce feature-level perturbations. Consistency constraints are applied between the probability outputs and soft pseudo-labels generated by these perturbations, significantly improving the model’s robustness.
(2) Based on the multi-branch decoder, we propose a contrastive learning strategy that models features in regions where predictions from the edge enhancement branch diverge from those of other branches. By reducing the discrepancy between predictions in these regions within the feature space, the strategy enhances segmentation performance, particularly in challenging areas such as burn edges.
(3) We developed an uncertainty correction mechanism that calculates the deviation between each branch’s prediction and the mean prediction, determining uncertainty with a single forward pass. This mechanism adaptively weights the consistency loss based on branch uncertainty, reducing the influence of unreliable predictions and better guiding the model’s training process.
(4) We evaluated our model on burn datasets with varying proportions of labeled and unlabeled data, and we compared its performance against several state-of-the-art (SOTA) methods. Experimental results demonstrate that SBCU-Net achieves superior performance in burn depth segmentation, surpassing existing SOTA approaches.

The structure of the remaining content of this article is as follows: Section 2 describes the related works on burn image segmentation and semi-supervised learning. Section 3 introduces the model structure and the design of the loss function. Section 4 describes the details and results of the experiment. Finally, Section 5 and Section 6 include discussions and conclusions, respectively.

## 2. Related Works

### 2.1. Main Models in the Field of Burn Image Segmentation

With the continuous advancements in deep learning technologies, convolutional neural networks (CNNs) have become a cornerstone in medical image segmentation. Among the most influential architectures, U-Net [13] stands out for its U-shaped encoder–decoder structure, which has been widely adopted for tasks such as organ segmentation, lesion detection, and cell tracking. Building upon U-Net, Sharp U-Net [23] introduces sharp blocks with depthwise separable convolutions before merging encoder and decoder features, thereby enhancing efficiency and multi-scale feature extraction for SOTA biomedical image segmentation. These advancements have demonstrated the potential of CNNs to handle complex medical imaging challenges. Inspired by the success of CNNs in medical image segmentation, researchers have increasingly explored their applications in burn diagnosis. For example, Liang et al. [24] proposed a U-shaped peak neural network inspired by retinal ganglion cells for burn and non-burn area segmentation, achieving a lightweight structure with a pixel accuracy of 92.89%. Chauhan et al. [25] introduced an atrous convolution-based network that leverages low- and mid-level features of the ResNet101 network [26] to extract rich semantic information, effectively segmenting burn areas from patient images. Liu et al. [17] integrated HRNet [18] into an end-to-end deep learning framework named HRNetV2-C1 for burn area segmentation and burn depth diagnosis, which is capable of distinguishing burn/non-burn areas and can attempt segmenting different burn depths. Although CNNs have demonstrated effectiveness in burn diagnosis, their inherent limitation in capturing contextual information due to the restricted receptive field of convolutional operators remains a challenge [27]. To tackle this problem, researchers have employed strategies to enlarge the receptive field and model long-range dependencies. For instance, Chang et al. [12] experimented with models such as U-Net [13], PSPNet [14], DeepLabV3+ [15], and Mask R-CNN [16]. Their findings indicated that DeepLabV3+ and PSPNet perform better in burn area segmentation by effectively expanding the receptive field. However, despite achieving relatively high segmentation accuracy in most cases, these approaches remain limited in capturing relationships between arbitrary locations. This limitation hinders further improvements in segmentation accuracy when dealing with complex or large burn wounds.

Unlike conventional CNNs, Transformers employ self-attention to model long-range relationships. This capability has been widely demonstrated in computer vision through successful models like DETR [28], Vision Transformer (ViT) [29], and SETR [30]. Leveraging these advancements, researchers have explored their potential in medical image segmentation, proposing models such as TransUnet [31], Swin-Unet [32], and BAT [33]. Our previous work proposed a joint-task framework that integrates a non-local network [34] to model long-range dependencies for burn depth segmentation combined with human body part detection to provide additional contextual guidance [35]. This framework not only improves the accuracy of burn depth segmentation, but it also enables a more precise estimation of burn area proportions. However, Transformer-based models often struggle to capture local information effectively. To address this, hybrid approaches such as TransFuse [36] and FAT-Net [37] integrate multiple encoders to combine global and local features. While these strategies aim to enhance feature representation, they can result in information mismatches during feature fusion, reducing fusion efficiency and limiting the ability to extract global information. Furthermore, the complex structures of these models significantly increase the parameter count and computational requirements, making training more challenging and restricting deployment on low-power devices like smartphones.

To address these challenges, our previous work proposed LTB-Net [22], utilizing the CSWin Transformer in its encoder to effectively capture long-range dependencies in burn images through cross-shaped window self-attention. Building on this, we introduced a lightweight local information enhancement module (LIEM) to capture local details of various sizes and a background information filtering module (BIFM) to accurately identify small burn areas by extracting cleaner background features from deep layers. Additionally, a layer-by-layer feature fusion multi-layer perceptron (MLP) decoder was designed to efficiently integrate multi-level features while reducing spatial and temporal complexity. Despite these advancements, LTB-Net relies on supervised methods that require high-quality labels and diverse datasets, necessitating expert annotations from burn specialists—a complex and resource-intensive process. To mitigate this dependency, research is increasingly shifting toward semi-supervised learning approaches. By utilizing both labeled and unlabeled data, these methods aim to reduce reliance on extensive expert annotations while maintaining reliable model performance.

### 2.2. Semi-Supervised Learning

Supervised learning-based segmentation algorithms have demonstrated their effectiveness in enhancing diagnostic accuracy and efficiency but are heavily dependent on extensive, high-quality, pixel-level labeled data for training. In medical image segmentation, obtaining such labeled data is often challenging and costly. Semi-supervised methods address this limitation by significantly reducing the reliance on labeled data, making them an increasingly popular research direction. Mainstream approaches in this domain include adversarial learning, pseudo-label learning, consistency regularization, and contrastive learning.

In adversarial learning, there are usually a generator and a discriminator. The generator generates data from unlabeled data, while the discriminator tries to distinguish between real data and generated data, thus facilitating the utilization of unlabeled data in semi-supervised learning. For example, Nie et al. [38] proposed an adversarial confidence network to help segmentation models utilize unlabeled data. Tang et al. [39] introduced a confidence map generated by a discriminator and added an auxiliary discriminator to mitigate the overfitting caused by limited labeled data. Pseudo-label learning trains a model on labeled data, uses it to predict unlabeled data to generate pseudo-labels, and then retrains the model on a combination of labeled and pseudo-labeled data. In pseudo-label learning, Bai et al. [40] employed an iterative update method using Conditional Random Fields to refine both network parameters and pseudo-labels. Peng et al. [41] proposed a collaborative training approach, where two models are trained simultaneously, encouraging consistent predictions on unlabeled data. Despite their advantages, pseudo-label learning methods risk undesirable segmentation results due to potential labeling errors, while adversarial learning often suffers from the unstable training that is caused by the interplay between the generator and discriminator.

Consistency regularization and contrastive learning have gained popularity for their robustness in semi-supervised methods. Consistency regularization assumes that when perturbations are applied to unlabeled data, the model’s prediction results should not change significantly. Tarvainen et al. introduced Mean Teacher [19], which enhances model generalization by minimizing the discrepancy between the outputs of student and teacher models. Yu et al. proposed UA-MT [21], which incorporates Monte Carlo dropout to introduce uncertainty and guide the model toward more reliable samples. MC-Net [20] and MC-Net++ [42] simulate network perturbations using multiple decoders and enforce prediction consistency by comparing their outputs. SS-Net [43] utilizes adversarial noise to impose pixel-level smoothness while encouraging inter-class separation. In contrast, contrastive learning compares the features of positive and negative samples, increasing the similarity among positive samples while decreasing the similarity between negative and positive samples. Therefore, researchers have introduced contrastive learning to semi-supervised tasks to extract more discriminative features. Zhong et al. [44] pioneered pixel-level contrastive learning with a negative sampling strategy for semantic segmentation. Zhao et al. [45] proposed a cross-layer contrastive learning framework to enhance the representation of local features.

However, existing methods like Mean Teacher [19] and MC-Net [20] apply perturbations in only one dimension, which limits their robustness. These approaches also lack specific optimization for burn edges, hindering their direct application in burn diagnosis. Furthermore, models such as UA-MT [21] estimate uncertainty through multiple forward passes, making training redundant and computationally expensive. To overcome these limitations, we present SBCU-Net, a semi-supervised burn depth segmentation network that introduces a multi-branch structure, integrates contrastive learning, and incorporates an uncertainty correction mechanism to address the challenges in burn diagnosis effectively.

## 3. Materials and Methods

### 3.1. Preliminaries

In semi-supervised segmentation methods, the training set typically includes labeled data and unlabeled data. For burn datasets with a combination of labeled and unlabeled data, the labeled datasets are denoted as $D_{l}=\left\{x_{l}^{i},y_{l}^{i}|i=1,...,N_{l}\right\}$, where $x_{l}^{i}$ represents the labeled input image, $y_{l}^{i}$ is the corresponding label, and $N_{l}$ represents the number of labeled samples. The unlabeled data set is represented as $D_{u}=\left\{x_{u}^{i},y_{u}^{i}|i=1,...,N_{u}\right\}$, where $x_{u}^{i}$ denotes the unlabeled input image and $N_{u}^{i}$ is the total number of unlabeled samples.

### 3.2. Framework Overview

**Figure 1:**
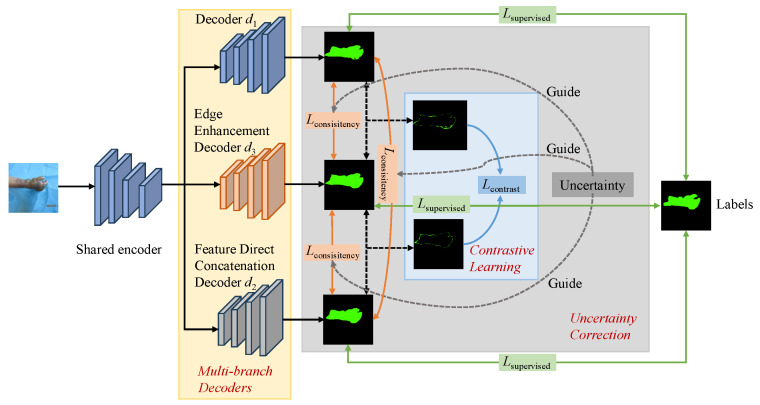
The overall structure of SBCU-Net, based on LTB-Net [22], adds the design of a multi-branch decoder, contrast learning, and uncertainty correction to make better use of unlabeled data.

The architecture of our proposed network is illustrated in Figure 1, and it consists of a shared encoder and three distinct decoder branches. One of these decoders is the decoder used in LTB-Net [22], while another employs feature fusion via direct feature concatenation to simulate small perturbations across different networks. The third decoder is an edge enhancement decoder, which aims to enhance the target edge areas by simulating erosion and dilation operations from morphological processing. This introduces additional perturbations from a feature perspective, improving the accuracy of edge segmentation results. The whole training process is divided into three main components: supervised training, consistency training, and contrastive learning. First, the labeled data $D_{l}$ is input into the network, where the three decoders generate different segmentation prediction maps. In this stage, the network calculates the supervised loss $L_{\mathrm{supervised}}$, which represents the difference between the segmentation prediction maps and the ground-truth labels, to conduct supervised training. Next, the unlabeled data $D_{u}$ is used, with consistency learning applied between the probability output of each decoder and the soft pseudo-labels generated by the other decoders to compute the consistency loss $L_{\mathrm{consistency}}$. In addition, the contrastive learning strategy is used to calculate the contrastive learning loss $L_{\mathrm{contrast}}$ for the regions where the prediction of the edge enhancement decoder branch is inconsistent with the other two branches so as to improve the segmentation effect of the burned edge. Finally, by calculating the difference between each branch probability output and the average of all branches probability output, the uncertainty is obtained and weighted to the $L_{\mathrm{consistency}}$ of the current branch to reduce the influence of outliers on consistent learning. Next, we introduced the complete process of our method, which encompasses the design of the decoder branches, the contrastive learning strategy, and the uncertainty correction mechanism.

### 3.3. Multi-Branch Decoders

#### 3.3.1. Feature Direct Concatenation Decoder

**Figure 2:**
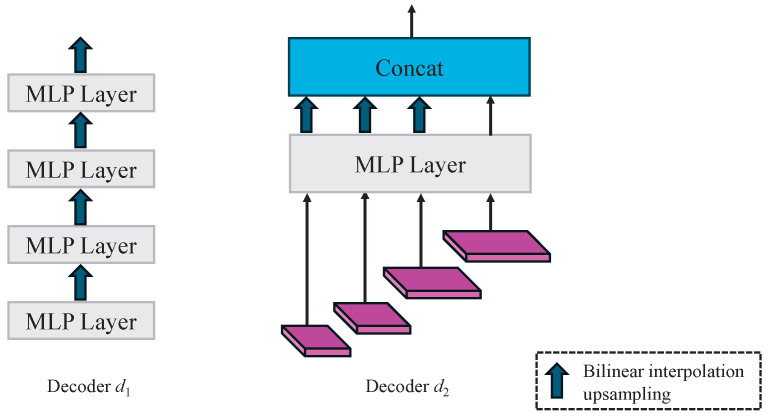
Different feature fusion methods of decoders. Decoder $d_{1}$ employs layer-by-layer fusion, and decoder $d_{2}$ utilizes direct concatenation.

Supervised learning trains models by maximizing the alignment between predictions and ground-truth labels. In semi-supervised learning, where unlabeled data are abundant, most methods impose consistency constraints on outputs under various perturbations, creating a supervisory signal for unlabeled data. MC-Net [20], for example, employs decoders with different upsampling methods to enforce consistency through network perturbations. Inspired by MC-Net, we introduced an additional MLP decoder branch based on our previous work. However, we observed that different upsampling methods significantly impact decoder predictions. Notably, nearest-neighbor upsampling performs substantially worse than bilinear interpolation. This performance disparity introduces a strong perturbation, making it challenging to enforce meaningful consistency constraints. As a result, the diverse upsampling strategies employed in MC-Net are unsuitable for our model. To resolve this challenge, we design a decoder branch that directly concatenates features from different layers, avoiding the adverse effects of strong perturbations. As depicted in Figure 2, decoder $d_{1}$ is the decoder in LTB-Net, which combines features from various encoder layers layer by layer, while decoder $d_{2}$ directly concatenates features from different layers.

#### 3.3.2. Edge Enhancement Decoder

**Figure 3:**
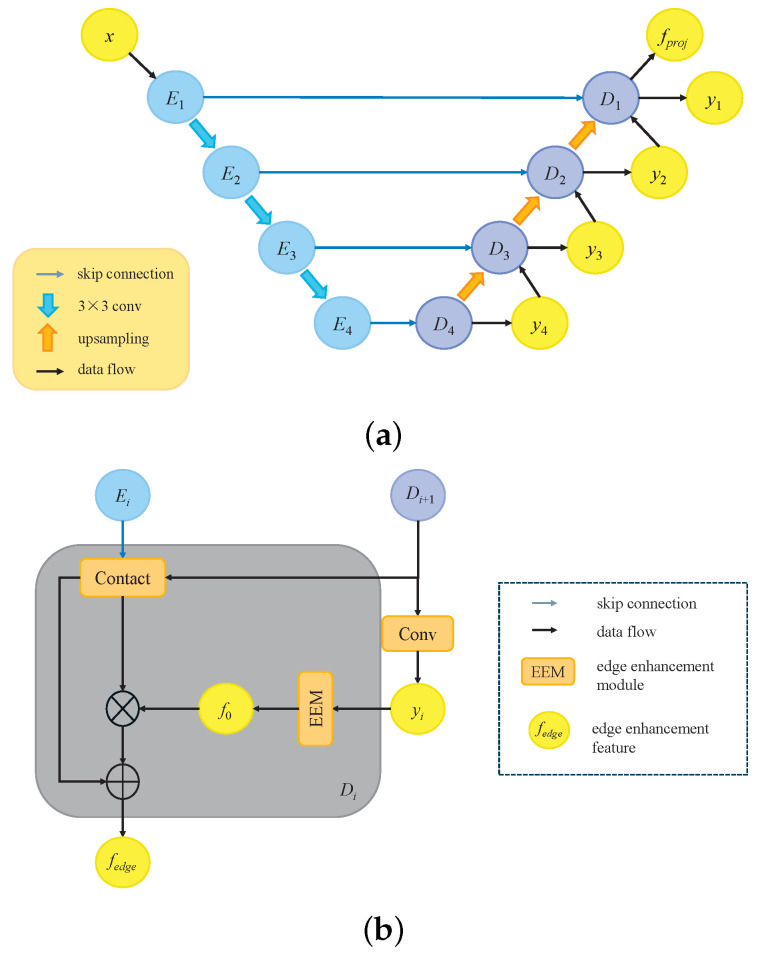
Structural introduction of a shared encoder and edge enhancement decoder: (**a**) general structure of a shared encoder and edge enhancement decoder; (**b**) detailed structure of an edge enhancement decoder.

Superficial burns often appear light red or pink, exhibiting low contrast against surrounding healthy skin. Additionally, burns can lead to localized edema, inflammation, or tissue damage, which blurs wound boundaries and complicates accurate segmentation. To address these challenges, inspired by MC-Net++ [42], we introduced an edge enhancement decoder as the third branch in our decoder network. Unlike MC-Net++, which incorporates perturbations in the decoder through different upsampling methods, our approach introduces feature-level perturbations by enhancing feature edges. This not only improves segmentation accuracy around burn edges, but it also leverages unlabeled data more effectively by regularizing perturbation consistency across different levels. The overall process of the shared encoder and edge enhancement decoder is illustrated in Figure 3a. The input features are first processed through a shared encoder, generating multi-level feature representations. These features are forwarded through skip connections to the edge enhancement decoder, enabling the decoder to capture refined features during the decoding process. Each decoder outputs a probability map representing the feature-level distribution within the image. The edge enhancement module extracts edge features from these probability maps, which are combined with skip-connected features to amplify edge details. The detailed design of the edge enhancement decoder is presented in Figure 3b.

Specifically, for features $f_{e}$ from the *i*-th layer encoder $E_{i}$, these are fused via skip connections with features $f_{d}$ from the decoder layer $D_{i+1}$. For $f_{d}$, a 3×3 convolution operation is applied to generate the corresponding probability map $y_{i}$, which can be described as follows:

(1) $$y_{i}={\mathrm{conv}}_{3\times 3}\left(f_{d}\right).$$

Then, $y_{i}$ passes through an edge enhancement module, which simulates the erosion and dilation operations in morphological processing, to obtain edge features. This process can be described by the following equation:

(2) $$f=softmax(y_{i}),$$

(3) $$f_{\min}=-maxpool\left(-f\right),$$

(4) $$f_{\max}=maxpool\left(f\right),$$

(5) $$f_{0}=\sum_{i=0}^{C}ReLU\left(f_{\max}-f_{\min}\right),$$

where *C* and $f_{0}$ denote the number of feature channels and the edge feature, respectively. Finally, residual connections are used to obtain the edge enhancement feature $f_{\mathrm{edge}}$, which can be represented as follows:

(6) $$f_{\mathrm{edge}}=concat\left(f_{e},f_{d}\right)+concat\left(f_{e},f_{d}\right)\cdot f_{0}.$$

### 3.4. Contrastive Learning Strategy

Contrastive learning is a strategy that enhances performance by minimizing the distance between positive sample pairs and maximizing the distance between negative pairs, effectively distinguishing the similarities and differences between samples. This method has been proven to significantly improve feature recognition in medical image segmentation. Unlike existing contrastive learning approaches that often rely on multiple modalities or multi-task learning [44,46,47], we implemented a contrastive learning strategy within a single modality using a multi-branch decoder architecture.

**Figure 4:**
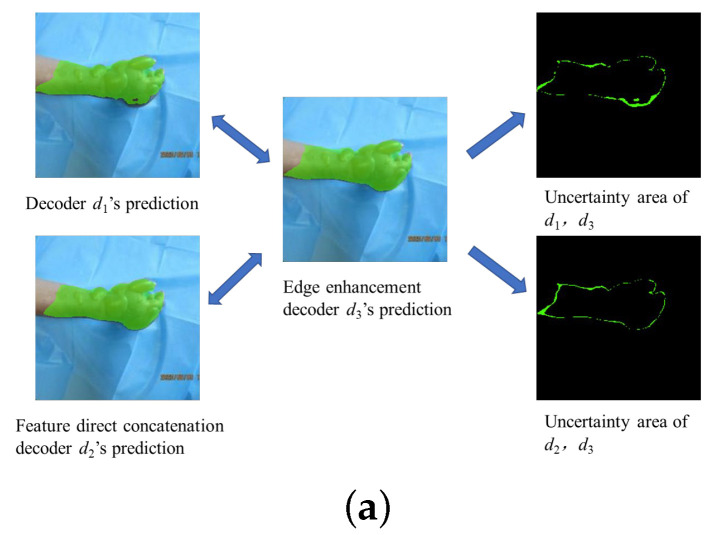

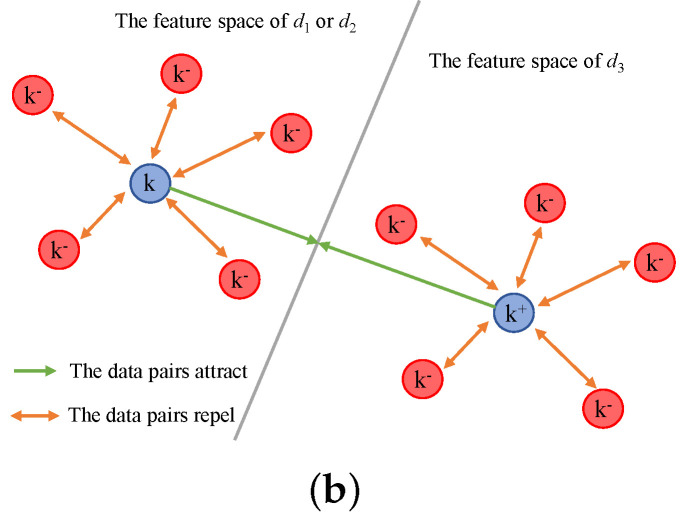
Contrastive learning process: (**a**) calculation of uncertainty regions; (**b**) positive and negative data pairs for contrastive learning.

As illustrated in Figure 4a, during the training phase, the areas where the predictions of the two decoders, $d_{1}$ and $d_{2}$, differed from the predictions of the edge enhancement decoder $d_{3}$ were considered uncertain regions. Therefore, to enhance the discrimination capability of $d_{1}$ and $d_{2}$ for these complex edge regions, we considered the pixels that were identically predicted by the two decoders and the edge enhancement decoder as positive samples, while the remaining pixels were treated as negative samples. This allowed us to apply a contrastive learning strategy to pull positive data pairs closer and push the negative data pairs apart in the feature space, as depicted in Figure 4b. The blue circles on the left represent the *k*-th pixel selected from either $d_{1}$ or $d_{2}$, the red circles denote negative samples, and the blue circles on the right represent the corresponding positive samples.

In terms of implementation details, taking decoders $d_{1}$ and $d_{3}$ as examples, the first step was to calculate the regions *K* where the predictions of the two decoders differed. The computation procedure can be formulated as follows:

(7) $$K=\{k|y_{1}^{k}\neq y_{3}^{k}\}.$$

In the equation, $y_{1}$ denotes the probability map generated by decoder $d_{1}$ and $y_{3}$ denotes the probability map generated by edge enhancement decoder $d_{3}$. Then, as shown in this section, a projection head was added to the final layer of the decoder. It comprised two 3 × 3 convolutional layers followed by a PReLU activation function. The process can be outlined as follows:

(8) $$f_{\mathrm{proj}}=conv_{3}\left(PReLU\left(conv_{3}\left(f_{d}\right)\right)\right),$$

where $f_{\mathrm{proj}}$ and $f_{d}$ denote the output features of the projection head and the decoder, respectively. For the selection of positive and negative samples, we randomly selected *N* points from the inconsistent region *K* as anchor points. For each anchor point *k*, the positive sample $k^{+}$ corresponds to the *k*-th pixel at the same position from the other decoder, while the negative sample $k^{-}$ refers to all other pixels in the anchor points except for the *k*-th pixel. Then, by calculating the similarity between positive and negative samples, the contrastive learning loss is derived. The calculation formula is as follows:

(9) $$L_{\mathrm{contrast}}=\frac{1}{N}\sum_{k=1}^{N}-\log\frac{e^{cos(k,k^{+})/\tau }}{e^{cos(k,k^{+})/\tau }+\sum_{k^{-}\in K^{-}}e^{cos(k,k^{-})/\tau }},$$

where cos denotes the cosine similarity and τ is the temperature parameter. The cosine similarity is defined by the following equation:

(10) $$cos\left(k,k^{+}\right)=\frac{f_{\mathrm{proj}}^{k}\cdot f_{\mathrm{proj}}^{k^{+}}}{∥f_{\mathrm{proj}}^{k}∥∥f_{\mathrm{proj}}^{k^{+}}∥}.$$

### 3.5. Uncertainty Correction

The multi-branch decoders utilize unlabeled data by reducing the discrepancy between their probability maps and the soft pseudo-labels produced by other decoders. However, with limited labeled data, some decoders may produce predictions that significantly deviate from the average, often indicating low reliability. Applying consistency constraints directly to these unreliable predictions risks misguiding the model training process. To overcome this challenge, we introduced an uncertainty correction mechanism.

In particular, we measured uncertainty using the KL divergence between the average prediction $p_{\mathrm{avg}}$ and the decoder *d*’s prediction $p_{d}$:

(11) $$U_{d}^{i}=\sum_{j=0}^{C-1}p_{d}^{i,j}\cdot \log\left(\frac{p_{d}^{i,j}}{p_{\mathrm{avg}}^{i,j}}\right).$$

In the equation, $U_{d}^{i}$ denotes the uncertainty of the pixel *i* output by the decoder *d*; *C* represents the number of segmentation categories in the task; and $p_{d}^{i,j}$ indicates the probability that the pixel *i* in the prediction output by decoder *d* belongs to class *j*. Uncertainty approximates the pixel-level difference between the decoder’s output $p_{d}$ and the average prediction $p_{\mathrm{avg}}$. For a given pixel, a larger $U_{d}^{i}$ means that the prediction for this pixel in decoder *d* deviates significantly from those of other decoders, implying high uncertainty. Consequently, by performing the above computation, we obtained a set of uncertainty maps $\left[U_{1},U_{2},U_{3}\right]$ for the three decoders, which reflect their respective uncertainties. According to the strategy in reference [48], the uncertainty value $w_{d}^{i}$ corresponding to the prediction of pixel *i* in decoder *d* can be obtained as follows:

(12) $$w_{d}^{i}=e^{-U_{d}^{i}}.$$

Next, to emphasize the reliable parts of the semi-supervised training predictions and disregard the unreliable ones, the estimated uncertainty was employed to automatically identify reliable pixels for loss computation:

(13) $$L_{\mathrm{uc}}=\frac{1}{D}\sum_{d=1}^{D}\frac{\sum_{i}w_{d}^{i}\sum_{j\neq d}MSE\left(p_{d}^{i},sp_{j}^{i}\right)}{\sum_{i}w_{d}^{i}}.$$

In the equation, *D* represents the number of decoders, MSE denotes the mean squared error, $p_{d}^{i}$ denotes the predicted pixel *i* by decoder *d*, and $sp_{d}^{i}$ denotes the soft pseudo-label for pixel *i* by decoder *d*. The relationship between soft pseudo-labels and decoder predictions is as follows:

(14) $$sp=\frac{p^{\frac{1}{T}}}{p^{\frac{1}{T}}+{\left(1-p\right)}^{\frac{1}{T}}},$$

where *T* denotes the constant controlling the sharpening temperature. Soft pseudo-labels aid in the entropy regularization of training [49], and, compared to the pseudo-labels generated by a fixed threshold, they can eliminate the influence of mislabeled training data [50].

From Equation (13), it can be seen that, for a given pixel from the decoder *d*, the higher the uncertainty, the lower the weight. Unlike many threshold-based methods [21,51], this strategy does not require additional manual operations to carefully design or adjust thresholds for different domains and can be accomplished with a single forward pass. Moreover, the study by Zheng et al. [48] indicates that the regularization term can prevent persistent high uncertainty during training, which may cause the model to ignore most samples. Therefore, we introduced the regularization term, which can be described as follows:

(15) $$L_{\mathrm{rt}}=\frac{1}{D}\sum_{d=1}^{D}U_{d},$$

where *D* represents the number of decoders.

### 3.6. Loss Function

The loss function consists of the supervised loss $L_{\mathrm{supervised}}$, consistency regularization loss $L_{\mathrm{consistency}}$, and contrastive learning loss $L_{\mathrm{contrast}}$, which can be described as follows:

(16) $$L=L_{\mathrm{supervised}}+\lambda L_{\mathrm{consistency}}+\lambda L_{\mathrm{contrast}},$$

where λ is the weight factor, and, in this case, it is a time-dependent Gaussian warming up function, defined as

(17) $$\lambda \left(t\right)=w_{\max}\cdot \exp\left(-5{\left(1-\frac{t}{t_{\max}}\right)}^{2}\right).$$

In the equation, $w_{\max}$ represents the final regularization weight, *t* represents the current training step, and $t_{\max}$ represents the maximum training step. For labeled data, $L_{\mathrm{supervised}}$ combines the cross-entropy loss $L_{\mathrm{CE}}$ and the dice loss $L_{\mathrm{Dice}}$, which is expressed as follows:

(18) $$L_{\mathrm{supervised}}=0.6\cdot L_{\mathrm{CE}}+0.4\cdot L_{\mathrm{Dice}}.$$

For unlabeled data, $L_{\mathrm{consistency}}$ is defined as

(19) $$L_{\mathrm{consistency}}=\alpha \cdot L_{\mathrm{uc}}+\left(1-\alpha \right)\cdot L_{\mathrm{rt}},$$

where α serves as a weighting factor to balance the contributions of the two terms, $L_{\mathrm{uc}}$ and $L_{\mathrm{rt}}$. For unlabeled data, by combining these two loss functions, the segmentation network can focus on reliable regions. This aids in minimizing the model’s overall uncertainty and leads to more consistent predictions across different decoders.

The contrastive loss $L_{\mathrm{contrast}}$ is used for all training data, including both labeled and unlabeled data. It is the sum of the contrastive losses between two different feature fusion decoders and the edge enhancement decoder:

(20) $$L_{\mathrm{contrast}}=L_{\mathrm{contrast}}^{d_{1}}+L_{\mathrm{contrast}}^{d_{2}},$$

where $L_{\mathrm{contrast}}^{d_{1}}$ and $L_{\mathrm{contrast}}^{d_{2}}$ represent the contrastive losses between the different feature fusion decoders $d_{1}$, $d_{2}$, and the edge enhancement decoder $d_{3}$.

## 4. Experiments

### 4.1. Dataset

At present, most datasets in burn research are obtained through cooperation with hospitals or are collected online [17,25,52,53]. For example, Liu et al. [17] worked with the The People’s Hospital of Jianggan District, Hangzhou, to obtain 516 unprocessed burn wound images that had been captured using smartphones and cameras, which were then annotated. Chauhan et al. [25] utilized a semi-automated script to search for keywords such as “burn injuries” and “partial thickness burns” on the Google search engine, collecting 449 burn images, and they collaborated with hospital burn experts to perform pixel-level annotations of the burn areas. However, due to data privacy and confidentiality, there are currently no publicly available burn depth segmentation datasets. Therefore, in order to meet the demand for high-quality, large-scale burn depth datasets, as utilized in our previous studies [22,35], we collected burn images taken with several different models of smartphones at hospitals between 2014 and 2022. After strict screening, images with unclear burn wounds due to factors such as illumination and wound occlusion were excluded. Subsequently, three burn experts used the LabelMe [54] annotation tool to annotate the images at the pixel level and retained data with consistent annotations. The dataset used in this work, which was also used in all previous studies, contained a total of 1,142 annotated images after removing duplicates. We divided the training, validation, and test sets in a ratio of 8:1:1, that is, the training set contained 914 images and the validation set and test sets each contained 114 images. It is worth noting that the main manifestations of hospital burn patients are superficial partial thickness and deeper burn depth. Considering the clinical research value, our burn depth classification is limited to superficial partial thickness (ST), deep partial thickness (DT) and full thickness (FT), in which the data volumes of ST, DT, and FT were 410, 554, and 178, respectively.

### 4.2. Experimental Environment

The model was built using version 1.8.0 of the PyTorch framework with CUDA version 11.8, and it was trained on an Ubuntu 16.04 system with an NVIDIA RTX 3090 Ti GPU (NVIDIA, Santa Clara, CA, USA). All input data were scaled to a size of 224 × 224 prior to being input into the network. Both labeled and unlabeled data had a batch size of 4. In addition, to enhance data diversity and reduce overfitting, we applied various data augmentation methods to the input training set, including scaling, random rotations of 45 degrees, random cropping, and random horizontal and vertical flipping. The network was trained with an AdamW optimizer with a learning rate of 0.0001 and momentum set to 0.9. Based on the experience of previous research [21], we also set $w_{\max}$ to 0.1. The backbone network was pretrained on the ImageNet dataset [55], and then the network was fine tuned for 100 epochs on the burn depth dataset. To prevent overfitting, we set a stopping criterion. Specifically, if the evaluation metrics on the validation set do not increase for more than three consecutive epochs, the training will be stopped in advance.

### 4.3. Experimental Results Analysis

To rigorously verify the effectiveness of the proposed semi-supervised learning approach, we conducted extensive comparisons with several SOTA approaches under two distinct levels of labeled data availability. The methods included Mean Teacher (MT) [19], Uncertainty-Aware Mean Teacher (UA-MT) [21], MC-Net [20], MC-Net++ [42], and SS-Net [43]. Simultaneously, our previous model, LTB-Net [22], served as the baseline to provide a consistent evaluation foundation. To ensure fair and meaningful comparisons, each semi-supervised algorithm was re-implemented within the LTB-Net framework and adopted the same fine-tuning method and stopping criteria as SBCU-Net, allowing us to accurately assess the performance and robustness improvements across models. Evaluation metrics include pixel accuracy (PA), the mean intersection over union (mIoU), the dice coefficient (DC), and 95% Hausdorff Distance (95HD). We also computed the DC for each depth class to evaluate the performance of different burn depths.

#### 4.3.1. Comparative Experiments with 50% Labeled Data

To assess the effectiveness of our approach in leveraging both labeled and unlabeled data, we performed comparative experiments using a dataset with 50% labeled and 50% unlabeled samples. As presented in Table 1, our method consistently achieved a superior performance across PA, mIoU, DC, and 95HD, exceeding the second-best method by 0.34%, 1.72%, 1.27%, and 1.15, respectively. While the DC score for DT was slightly lower than the supervised model, our method outperformed the supervised LTB-Net across all other metrics, achieving significant improvements of 5.44% in mIoU, 4.67% in DC, 5.17 in 95HD, and an impressive 17.43% in DC for FT. These results highlight our approach’s capacity to effectively leverage unlabeled data, substantially enhancing burn depth segmentation performance.

**Figure 5:**
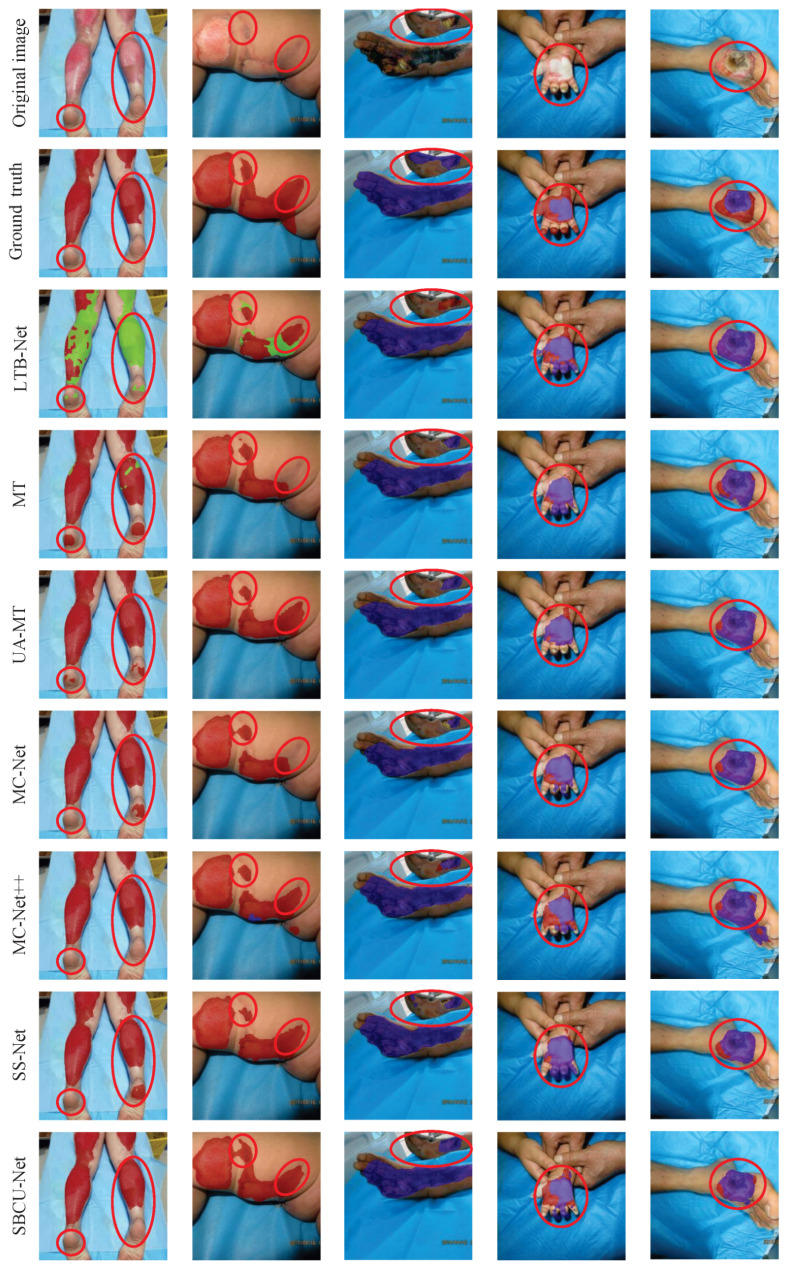
Visualization of the experimental results of the different models with 50% labeled data. The red circular box indicates areas where our method showed significant improvement in burn depth segmentation. The green, red, and blue covered areas represent ST, DT, and FT burn depths, respectively.

For a more intuitive comparison, Figure 5 presents visual results of all the evaluated methods. The semi-supervised learning methods consistently showed better segmentation performance compared to supervised learning, highlighting the potential of leveraging unlabeled data. Among these, our approach achieved the finest and most accurate segmentation details, particularly in challenging edge areas. For example, in the second column, our method delivered precise contour segmentation, outperforming other semi-supervised approaches. In the third column, where other methods misclassified or overlooked portions of FT in the right foot region, our approach significantly improved segmentation accuracy, capturing complex and subtle features effectively.

#### 4.3.2. Comparative Experiments with 10% Labeled Data

To further assess the performance and robustness of SBCU-Net with less labeled data, we conducted quantitative comparisons across all methods using only 10% labeled samples. Table 2 shows that SBCU-Net consistently outperforms other semi-supervised methods on nearly all metrics. Compared to the supervised baseline, SBCU-Net achieves significant gains of 1.94% in PA, 6.50% in mIoU, 5.60% in DC, and 8.56 in 95HD. It also exhibited notable improvements in DC across burn depths, with increases of 9.52% for ST, 6.50% for DT, and 5.95% for FT. These results highlight SBCU-Net’s effectiveness in leveraging unlabeled data to enhance segmentation performance, especially in scenarios with limited labeled data. In contrast, methods like MT and MC-Net demonstrate only marginal improvements over the supervised baseline, suggesting that some semi-supervised approaches may generate inaccurate predictions on unlabeled data when limited labeled samples are available. Without proper filtering, these predictions can negatively impact consistency loss and degrade performance. SBCU-Net effectively addresses this issue through an uncertainty correction mechanism that weights consistency loss, significantly enhancing overall performance.

**Figure 6:**
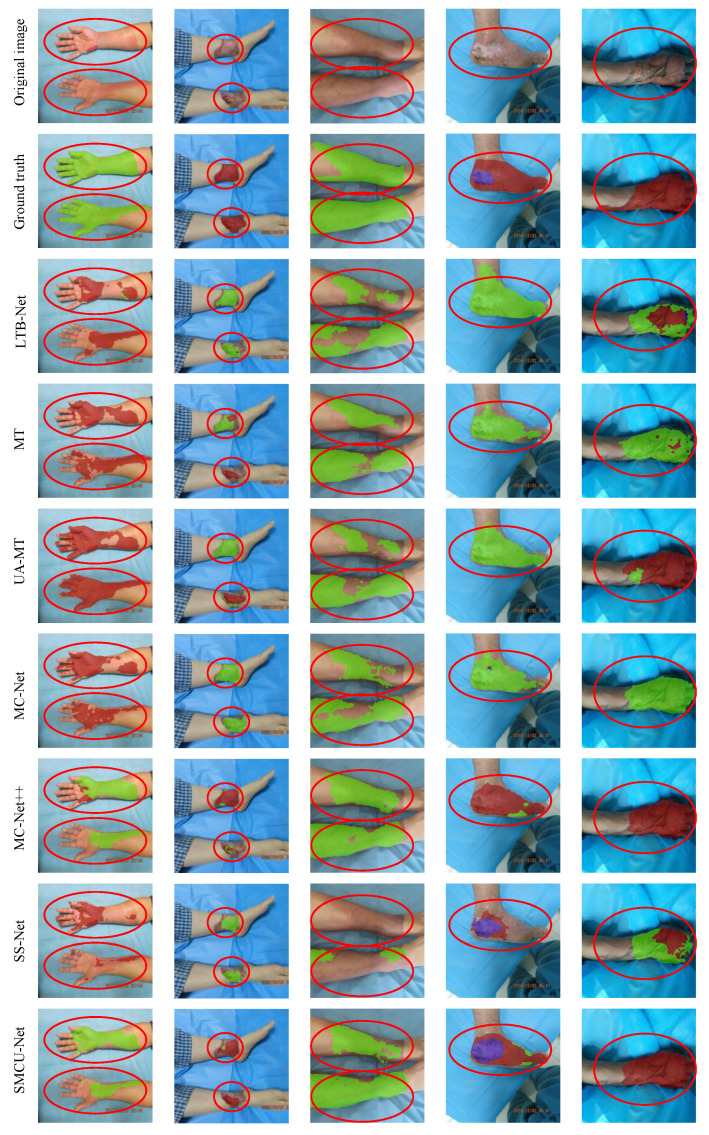
Visualization of experimental results of different models with 10% labeled data. The red circular box indicates areas where our method showed significant improvement in burn depth segmentation. The green, red, and blue covered areas represent ST, DT, and FT burn depths, respectively.

Figure 6 illustrates visual results with 10% labeled data, demonstrating SBCU-Net’s ability to accurately segment burn regions and depths even under constrained conditions. For instance, in the third column, both supervised and other semi-supervised methods failed to distinguish ST regions from normal skin, particularly in large ST areas. SBCU-Net, leveraging consistency constraints across multiple perturbation levels, effectively captured finer features. In the fourth column, SBCU-Net accurately identifies DT and FT regions, where other methods misclassified them as ST. These visual comparisons further underscore SBCU-Net’s superiority in challenging segmentation tasks.

### 4.4. Ablation Study

To comprehensively evaluate the contributions of each sub-module in the proposed SBCU-Net, ablation experiments were performed with 50% and 10% labeled data. These experiments reveal the individual and collective effects of each module on segmentation accuracy and model robustness. Additionally, the influence of the number of decoder branches on the overall results was investigated, where the aim was to balance efficiency and accuracy.

#### 4.4.1. Module Ablation Experiment

The proposed SBCU-Net introduces several key innovations, including the feature direct concatenation decoder, the edge enhancement decoder, the contrastive learning strategy, and the uncertainty correction mechanism. To assess the effectiveness of each improvement, we employed the dual decoder branch structure, which consists of nearest neighbor interpolation and bilinear interpolation as the baseline method, and we conducted a series of ablation experiments on 50% labeled data and 10% labeled data. The results are shown in Table 3 and Table 4, respectively. As can be seen from the table, under different proportions of unlabeled data, after replacing the nearest neighbor interpolation upsampling decoder of the baseline method with our feature direct concatenation decoder, both mIoU and DC were improved, which confirms that the strong perturbation brought by the nearest neighbor upsampling decoder is not conducive to consistency constraints, while the feature direct concatenation decoder can well circumvent this problem. In addition, the introduction of the edge enhancement decoder enables the model to achieve stable performance improvements on different proportions of labeled data, indicating that, by introducing feature-level perturbations to the edge enhancement of features, the consistency constraints between different branches and the robustness of the model can be further enhanced. Combined with the contrastive learning strategy to further process the edge regions, PA, mIoU, DC, and 95HD were improved by 0.23%, 0.77%, 0.55%, 2.38, and 0.72%; and 0.64%, 0.46%, and 2.17 under the 50% and 10% labeled data, respectively. The evident improvement of 95HD further proves the effectiveness of the edge enhancement decoder and contrastive learning strategy for improving edge region segmentation. It is worth noting that, when uncertainty correction is applied, the performance improvement of 10% labeled data is more evident than that of 50% labeled data, with PA, mIoU, and DC increasing by 0.53%, 1.46%, and 1.06%, respectively. This confirms that using uncertainty correction to weight the loss can effectively mitigate the impact of unreliable predictions on model training when labeled data are limited.

#### 4.4.2. Ablation Experiment on Decoder Branches

**Figure 7:**
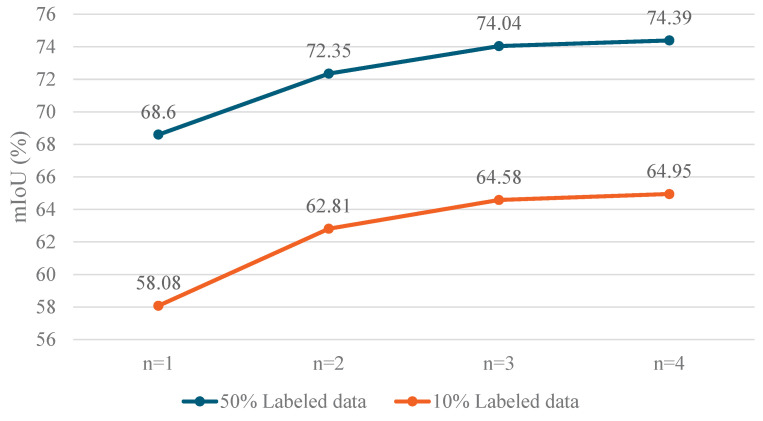
The impact of different numbers of decoder branches on the results.

The number of decoders *n* is designed to be scalable. To examine how the number of decoder branches affects model performance, we conducted an ablation experiment where decoders were added sequentially: first, the feature layer-by-layer fusion decoder, and this was followed by the feature direct concatenation decoder, the edge enhancement decoder, and, finally, the feature layer-by-layer fusion decoder with different initialization methods. The results of these experiments are presented in Figure 7, which demonstrate that adding more decoders generally improves model accuracy. However, when n=3, the performance improvement becomes marginal. Specifically, with 50% and 10% labeled images, the improvement with the addition of further decoders is only 0.35% and 0.37%, respectively. This suggests that, with limited labeled data, deep models may generate high-confidence but incorrect predictions, which can limit the effectiveness of adding more decoders. This phenomenon aligns with findings from previous studies, such as in [56]. Therefore, considering the trade off between model efficiency and accuracy, we determined that setting n=3 provides the optimal balance for SBCU-Net.

## 5. Discussion

### 5.1. Effects of Hyperparameter α

**Figure 8:**
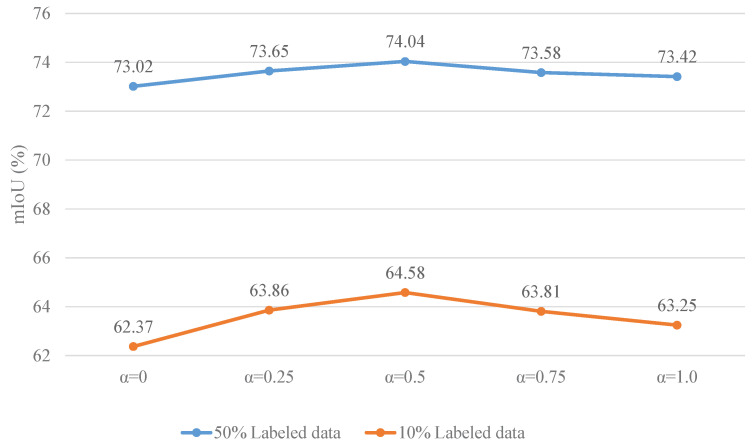
Performance of our model with different α.

We conducted a sensitivity experiment on the hyperparameter α involved in Equation (19) to determine the most appropriate weight for $L_{\mathrm{uc}}$ and $L_{\mathrm{rt}}$ in $L_{\mathrm{consistency}}$. We set α to 0.0, 0.25, 0.5, 0.75, and 1.0, and the experimental results under 50% and 10% labeled data are shown in Figure 8. From the figure, it can be observed that a smaller α does not sufficiently perform uncertainty correction, making it difficult for the model to further filter out inaccurate predictions. On the other hand, a larger α, due to consistently high uncertainty, ignores a majority of the samples, which also leads to a decline in model performance. Therefore, we set α to 0.5.

### 5.2. Future Works

Burn depth diagnosis is a critical aspect of clinical burn management, and advancements in this area can significantly enhance patient outcomes. SBCU-Net, by integrating multi-branch decoders, contrastive learning strategies, and uncertainty correction, addresses key challenges such as data scarcity, annotation difficulties, and edge segmentation accuracy. Its ability to achieve precise segmentation, especially in edge regions critical for burn severity assessment, demonstrates the potential of semi-supervised approaches in this field. To our knowledge, this is the first attempt to apply a semi-supervised framework specifically for burn depth segmentation, offering a promising foundation for further exploration in this domain.

Despite its achievements, there are numerous opportunities for improvement and future research. Firstly, there are currently no publicly available datasets in the field of burn depth segmentation. The use of a single internal dataset may lead to limitations in the research results and raise concerns about generalizability. In the future, we will actively seek more diverse data sources, including potential public datasets and collaborations with additional hospitals to expand the scope of data collection. This will further validate and enhance the reliability and generalizability of the model. Second, the multi-branch decoder of SBCU-Net was designed based on an MLP decoder architecture, making it particularly well suited for Transformer-based frameworks. In future research, we will explore more generalizable designs that enable the model to flexibly adapt to different network architectures while maintaining robust performance. In addition, we observed that, when the unlabeled data are small (50%), the DC score of DT is slightly lower than that of the supervised model. We speculate that the main reason for this is because the features of DT are complex and transitional. The relatively deep ST and DT wounds exhibit a high degree of similarity, and they can only be distinguished by minor changes such as dryness. There are differences in the feature extraction and fusion methods between the decoder of a different structure and the decoder of the supervised model. When the proportion of unlabeled data is small, the decoder of a different structure may extract feature representations that do not match those of the decoder of the supervised model. When these mismatched features are fused, it may lead to information confusion, causing the model to deviate in classifying the DT and ST areas. This reduces the positive gain brought by the information of unlabeled data, slightly decreasing the DC score of DT. To address this limitation, in the future, we will start from how to better extract different decoder features to avoid information confusion caused by mismatched features of different decoders.

Another critical challenge lies in the computational demands of burn image segmentation. Deploying SBCU-Net for real-time clinical assistance, such as on mobile devices, requires further optimization to reduce computational complexity without sacrificing accuracy. Lightweight models or hardware-specific optimizations could enable effective deployment on portable diagnostic tools. Furthermore, clinical burn treatment requires not only precise depth diagnosis, but it also requires an accurate estimation of the burn surface area. In a previous work [35], we integrated body part detection methods to estimate the percentage of total body surface area (TBSA) affected by burns. Future efforts will extend this by utilizing data with ruler references to estimate the actual burn area more accurately, combining depth and surface area measurements for a comprehensive diagnostic solution.

By overcoming these challenges and broadening the current framework, we aim to contribute to the broader application of AI in burn care, ensuring both diagnostic accuracy and clinical practicality.

## 6. Conclusions

This work proposes SBCU-Net, a semi-supervised burn depth segmentation network with contrastive learning and uncertainty correction, to explore the application of semi-supervised methods in burn depth segmentation. The network is based on the supervised model LTB-Net [22] from previous work and incorporates three core components: a multi-branch decoder, contrastive learning, and an uncertainty correction mechanism. The multi-branch decoder introduces perturbations from different dimensions through the feature direct concatenation decoder and the edge enhancement decoder, enabling the model to better perform consistency regularization under varying amounts of labeled data, thereby improving robustness. To enhance the model’s capability in segmenting edge regions, contrastive learning is applied between the edge enhancement decoder branch and the other two decoders in regions where predictions are inconsistent. Furthermore, an uncertainty correction mechanism is proposed, which differs from UA-MT [21] by not relying on Monte Carlo dropout or the determination of screening thresholds through multiple forward propagations. Instead, it automatically adjusts the consistency loss of decoder branches based on prediction uncertainty, making it more suitable for burn depth segmentation and more efficient in guiding the model’s training direction. Experimental results demonstrate that SBCU-Net outperforms existing methods in burn depth segmentation, particularly in edge regions, showcasing the potential of semi-supervised methods in this field. Future efforts will focus on further improving and applying the proposed method to promote advancements in burn depth segmentation research.

## Figures and Tables

**Table 1:** Comparison experiment results with 50% labeled data.

Method	PA	mIoU	DC	95HD	DC (ST)	DC (DT)	DC (FT)
LTB-Net [22]	93.98	68.60	79.84	12.34	78.25	83.28	61.16
MT [19]	93.48	70.27	81.64	11.58	70.17	80.78	77.94
UA-MT [21]	93.96	71.61	82.68	10.26	74.42	83.59	75.16
MC-Net [20]	93.97	70.98	82.16	12.15	74.37	**83.60**	72.99
MC-Net++ [42]	93.80	72.32	83.24	10.67	74.76	82.63	77.95
SS-Net [43]	93.25	72.16	83.14	8.32	73.42	80.33	**81.57**
SBCU-Net	**94.32**	**74.04 **	**84.51**	**7.17**	**78.68**	83.03	78.59

**Table 2:** Comparison experiment results with 10% labeled data.

Method	PA	mIoU	DC	95HD	DC (ST)	DC (DT)	DC (FT)
LTB-Net [22]	90.16	58.08	71.35	23.74	63.02	73.67	52.78
MT [19]	90.72	59.55	72.67	20.51	64.38	75.13	54.94
UA-MT [21]	91.04	60.23	73.35	19.68	62.16	74.46	60.09
MC-Net [20]	90.18	60.48	73.66	21.54	58.22	74.74	65.70
MC-Net++ [42]	91.10	62.35	75.28	20.61	60.60	76.53	67.83
SS-Net [43]	90.21	61.02	74.00	15.32	53.79	74.77	**71.91**
SBCU-Net	**92.10**	**64.58**	**76.95**	**15.18**	**72.54**	**80.17**	58.73

**Table 3:** Ablation experiment results of modules with 50% labeled data.

Baseline Method	Feature Direct Concatenation Decoder	Edge Enhancement Decoder	Contrastive Learning	Uncertainty Correction	PA	mIoU	DC	95HD
✔					93.97	70.98	82.16	12.15
	✔				93.81	71.82	82.87	11.98
	✔	✔			94.02	72.86	83.64	10.24
	✔	✔	✔		94.25	73.63	84.19	7.86
	✔	✔	✔	✔	**94.32**	**74.04**	**84.51**	**7.17**

**Table 4:** Ablation experiment results of modules with 10% labeled data.

Baseline Method	Feature Direct Concatenation Decoder	Edge Enhancement Decoder	Contrastive Learning	Uncertainty Correction	PA	mIoU	DC	95HD
✔					90.18	60.48	73.66	21.54
	✔				90.72	61.50	74.42	21.17
	✔	✔			90.85	62.48	75.43	19.36
	✔	✔	✔		91.57	63.12	75.89	17.19
	✔	✔	✔	✔	**92.10 **	**64.58**	**76.95**	**15.18**

## Data Availability

The data used to support the findings of this study are available from the authors upon request.
